# Molecular underpinnings of division of labour among workers in a socially complex termite

**DOI:** 10.1038/s41598-021-97515-w

**Published:** 2021-09-14

**Authors:** Daniel Elsner, Klaus Hartfelder, Judith Korb

**Affiliations:** 1 Evolutionary Biology & Ecology, University of Freiburg, Freiburg, Germany; 2 Faculdade de Medicina de Ribeirão Preto, Universidade de São Paulo, Ribeirão Preto, Brazil

**Keywords:** Evolution, Social evolution

## Abstract

Division of labour characterizes all major evolutionary transitions, such as the evolution of eukaryotic cells or multicellular organisms. Social insects are characterized by reproductive division of labour, with one or a few reproducing individuals (queens) and many non-reproducing nestmates (workers) forming a colony. Among the workers, further division of labour can occur with different individuals performing different tasks such as foraging, brood care or building. While mechanisms underlying task division are intensively studied in social Hymenoptera, less is known for termites, which independently evolved eusociality. We investigated molecular mechanisms underlying task division in termite workers to test for communality with social Hymenoptera. We compared similar-aged foraging workers with builders of the fungus-growing termite *Macrotermes bellicosus* using transcriptomes, endocrine measures and estimators of physiological condition. Based on results for social Hymenoptera and theory, we tested the hypotheses that (i) foragers are in worse physiological conditions than builders, (ii) builders are more similar in their gene expression profile to queens than foragers are, and (iii) builders invest more in anti-ageing mechanism than foragers. Our results support all three hypotheses. We found storage proteins to underlie task division of these similar-aged termite workers and these genes also characterize reproductive division of labour between queens and workers. This implies a co-option of nutrient-based pathways to regulate division of labour across lineages of termites and social Hymenoptera, which are separated by more than 133 million years.

## Introduction

Benefits associated with division of labour (DOL) are discussed as major driving forces favouring major transitions in evolution, such as the evolution of the eukaryotic cell or multicellular organisms^1^. The evolution of eusociality is often regarded as the latest of these major evolutionary transitions. Social insects offer the advantage to study ‘transition phases’ as not all social insects are superorganisms (i.e., species, in which the workers of a colony cannot reproduce and are sterile^2^), which would qualify as having accomplished a major evolutionary transition^2,3^. Social insects are also characterized by DOL, namely between reproductive and non-reproducing animals within an insect colony. In the social Hymenoptera, comprising ants and some bees and wasps, as well as in the Blattodean termites, only one or a few individuals within a colony reproduce (queens and in termites also a king), while the large majority of the individuals of a colony, forgo reproduction, at least temporarily, and perform worker or soldier tasks. Within the worker caste further DOL commonly exists that is associated with worker age (i.e., age polyethism)^4^. Young workers are performing tasks within the nest, such as caring for the brood and the reproductives, while older workers perform riskier tasks, such as foraging outside the nest^4^. Such age-based DOL is considered adaptive^5–7^ as older individuals generally have a reduced life expectancy, and thus a lower reproductive value (or its inclusive fitness equivalent) than young workers^8^.

Proximate molecular mechanisms underlying task division have been intensively studied in social Hymenoptera^9,10^. Vitellogenins (Vgs) have emerged as major regulators of DOL between queens and workers^11–15^, as well as between workers performing different tasks^13,16,17^. They are storage proteins that are well-known for their function as yolk precursors during oogenesis in female insects^18^. For instance, in the best studied social insect, the honeybee *Apis mellifera*, the transition from indoor nursing tasks to outdoor foraging is characterized by a switch in the expression of many genes^19^. Physiologically, the age-related switch from nursing to foraging is associated with an increase in the haemolymph juvenile hormone (JH) titre^9,20^ and triggered by a decrease in the circulating Vg levels^15,21–23^. Importantly, in honeybee workers, which do not produce eggs in the presence of the queen, Vg has been shown to have immunosenescence-inhibiting function^24^.

Compared to bees, much less is known about the molecular mechanisms underlying DOL, and especially task division among workers in termites. Termites (infraorder ‘Isoptera’, formerly considered an independent order^25^) are social cockroaches, a monophyletic lineage nested within the Blattodea that evolved eusociality independently from social Hymenoptera^25,26^. As in the social Hymenoptera, age polyethism is common with older workers performing riskier tasks such as foraging or defence^27,28^. It is also established (i) that Vgs play an important role in DOL between termite reproductives and workers^11,29,30^, (ii) that JH titres and Vg expression are positively associated in termite queens^31,32^, and (iii) that queens have considerable higher JH titres than workers^33–35^. Termites have three Vg copies that are upregulated in queens^36,37^ and at least in *Cryptotermes secundus* these three Vg copies are members of a gene coexpression network that characterizes the queen phenotype (see also “Discussion”)^30^. These results lead us to hypothesize that Vgs and JH not only underlie reproductive DOL but also regulate task division among termite workers, similar as in social Hymenoptera.

We aimed at testing whether task division among workers in termites is associated with the same mechanisms underlying task division in social Hymenoptera. To do this, we chose a termite species for which task division among workers has been intensively studied and is best described, *Macrotermes bellicosus* Smeathman (Macrotermitinae)^28,38,39^. *M. bellicosus* is a fungus-growing termite that builds large mounds and has a complex colony structure consisting of up to a few millions of individuals divided into four sterile castes: major and minor workers, and major and minor soldiers^40^. DOL among these sterile castes has been intensively studied, especially for minor workers^28,38,39^. They are females and thus can be compared with the queen. In addition, they make up the majority of individuals and have the most diverse behavioural repertoire. Young minor workers take care of the brood and queen. Old minor workers perform building and foraging. The latter allows to test causes of task division of similar-aged individuals, thus overcoming the confounding effect of age *versus* task, which can occur in studies addressing age-based task division (i.e., age polyethism). In addition, using similar-aged workers offers a rare opportunity to testing whether riskier tasks are associated with a reduced investment in anti-ageing mechanisms, regardless of intrinsic age. This is expected from life history theory, which predicts that age-dependent higher extrinsic mortality by random causes can select for faster intrinsic ageing (i.e. senescence) associated with less investment in anti-ageing mechanisms^41,42^. In *M. bellicosus*, all minor workers are females; males develop into major workers, which have completely different tasks within a colony and hence are not addressed in this study.

We tested the hypothesis that in minor workers (for simplicity, hereafter just workers) foragers (hereafter, foragers) are in worse physiological conditions than those that perform indoor construction tasks (hereafter, builders). Thus, we also hypothesized that builders are more similar to queens in their overall gene expression profiles than foragers. Finally, we tested whether builders invest more in anti-ageing mechanism than foragers, the latter facing higher age-specific extrinsic mortality risks.

To test these hypotheses, we generated transcriptomes of heads plus thorax (for rational of tissue choice, see Methods) from builders and foragers. After comparing gene expression between tasks of workers, we compared their expression profiles to that of queens, which have been published in an earlier study that investigated age- and caste-related gene expression differences in *M. bellicosus*^43^. To further characterize the physiological conditions of workers, we determined and compared the presence of urocytes in builders and foragers. Urocytes are one of three cell types in the fat body of termites; they are storage cells for nitrogenous waste products^44,45^. The accumulation of urocytes can be an indicator of the ‘unwell’-being in termites, though their role seems to be complex (for more details, see Discussion)^44–46^. Our study was complemented with endocrine measurements to compare whether foragers and builders differ in haemolymph JH titres.

## Results

### Comparison of gene expression between builders and foragers

Using a total of 14 head/thorax transcriptomes (builders: N = 7; foragers: N = 7), we found 939 differentially expressed genes (DEGs) between builders and foragers (for a full list of genes, see Supplementary Material). In builders, 593 genes were significantly overexpressed (Supplementary Table S1). Among the ‘builder genes’, all three genes encoding termite Vgs were especially highly expressed (log2FC > 2) (Supplementary Table S2). Also upregulated in the builders were five antioxidant genes: two genes encoding catalases (Cat) and three genes encoding superoxide dismutases (SOD) (2 × CuCn, 1 × MnFe types). The top enriched GO terms for builders were energy metabolism, specifically ATP metabolism and glycolytic process, as well as translation, ribosomal genes, and genes related to protein transport (Supplementary Table S3, Supplementary Figs. S1–S3).

Three hundred and forty-six genes were significantly overexpressed in foragers (Supplementary Table S4) but no striking patterns were apparent. Foragers were characterized by the following GO terms: protein kinase activity, heparin binding, regulation of transcription, RNA metabolism, protein phosphorylation and DNA polymerase activity (Supplementary Table S3, Supplementary Figs. S4–S6).

### Comparison of worker task-related gene expression with ‘queen genes’

**Figure 1:**
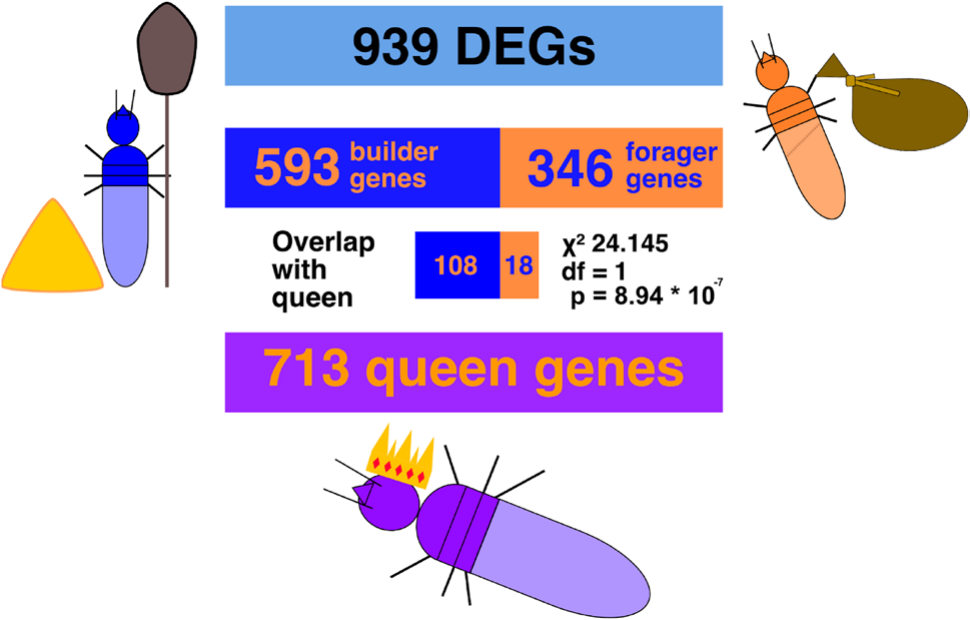
Graphical representation of overlapping genes. We compared the DEGs between builders and foragers (939) with genes that were upregulated in queens compared to the other castes (713). The data for the latter were derived from our previous study^43^. Of the DEGs, those upregulated in builders were more commonly shared with queens (108 of 593) than those upregulated in foragers (18 of 346) (χ^2^ = 24.15, df = 1, p = 8.94 × 10^–7^). Therefore, builders seem to be more queen-like in their gene expression than foragers.

To test whether genes upregulated in builders are more similar to genes highly expressed in queens, we compared the current task-specific expression differences of workers with the set of genes that are specifically upregulated in *M. bellicosus* queens compared to kings and workers (‘queen DEGs’ hereafter)^43^. In total 713 DEG were queen specifically expressed^43^. Out of the 593 genes that were upregulated in builders compared to foragers, 108 (18.2%) DEGs were queen DEGs. By contrast, of the 346 forager genes, only 18 (5.2%) DEGs overlapped with queen DEGs. Thus, builders shared significantly more genes with queens than foragers did (χ^2^ = 24.15, df = 1, p = 8.94 × 10^–7^). Among the genes that were shared between builders and queens were the three *vg* genes (Fig. 1).

### Presence of urocytes and JH titres in builders and foragers

**Figure 2﻿:**
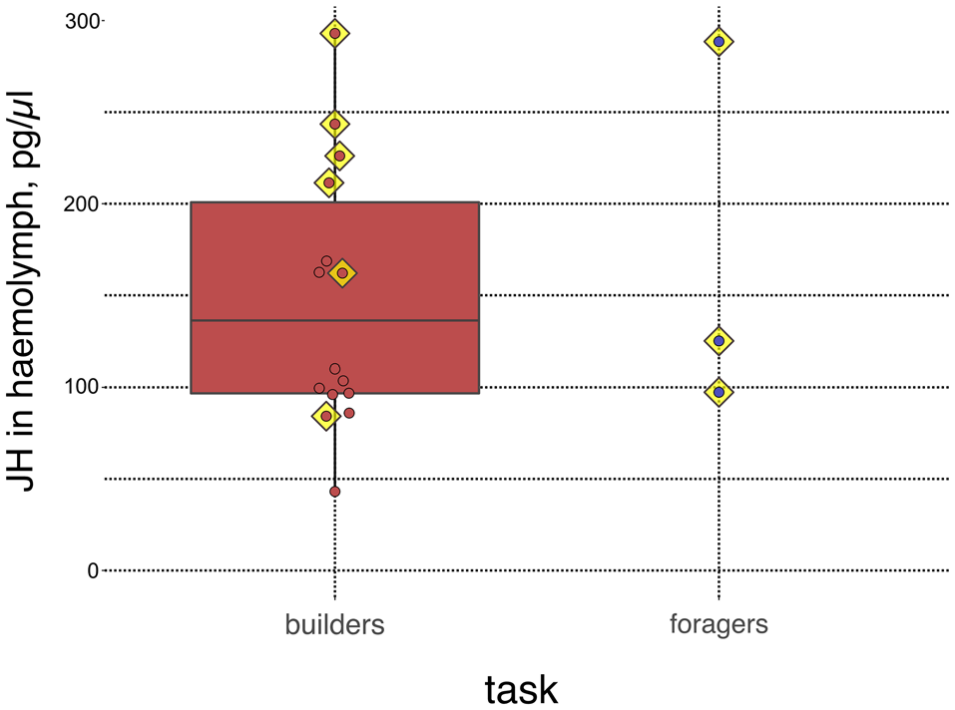
Boxplot of JH titres in builders compared to foragers. Builders and foragers did not differ significantly in their haemolymph JH titres (Mann Whitney U test: N = 17, U = 18.0, p = 0.705) (Supplementary Table S5). Box plot elements are defined as follows: Centre line: Median. Box range: 25 and 75 percent quartiles. Dots : Individual data points. Yellow squares under dots : Presence of uric acid. For the three data points of the foragers , no box was drawn as quartiles are not meaningful.

Although sample sizes are small, screening builders and foragers for the presence of urocytes implies a task effect. All three foragers had visible urocytes, while (only) 6 out of 14 builders had them (contingency analysis: χ^2^ = 3.24, P = 0.072; Supplementary Table S5). The effect size of this analysis was 0.44 and the power 0.45. By contrast, builders and foragers did not differ significantly in their haemolymph JH titres (Mann Whitney U test: N = 17, U = 18.0, p = 0.705) (Fig. 2).

## Discussion

### Vitellogenin, a commonality underlying division of labour across social insects

Similar to social Hymenoptera (e.g. honeybee^15,22–24,46,47^, ants^17^), we detected a downregulation in the expression of Vg genes in foraging termite workers compared to builders. This commonality of a co-option of Vg to regulate task division among workers across lineages, which are separated by more than 133 million years^48^, is striking. We studied head plus thorax tissues. Thus it might seem surprising to detect a Vg signal, which is linked to reproduction. Yet, in the termite *C. secundus*, at least one of these Vg genes, *Neofem3*, is similarly expressed in all body parts (head, thorax, abdomen)^29^. Furthermore, recent results demonstrate that head plus thorax tissues are very well suited to study life history traits^30,49^, including expression of all three Vg genes that we detected to be upregulated in builders compared to foragers. Fat body, the main tissue known to synthetize vitellogenin, also occurs in the thorax.

### Molecular mechanisms underlying task division reflect mechanisms underlying reproductive division of labour

Equally striking is the fact that vitellogenins also characterize reproductive DOL between queens and workers in social Hymenoptera and termites, with queens being characterized by high nutrient conditions^30,50^. The ovarian ground plan hypothesis^51^, and its reformulation as reproductive ground plan hypothesis^21,24,52^, state that molecular genetic modules regulating reproduction in solitary insects have been co-opted during social evolution to underlie reproductive DOL. Workers (physiologically) reflect the foraging and brood caring stage of females of non-eusocial insects, while the queens of social insects are physiologically equivalent to the reproducing solitary insects. Accordingly, reproductive DOL can be achieved through a decoupling and heterochronic shift in the expression of the corresponding genetic modules derived from solitary ancestors. There is support for this hypothesis in social Hymenoptera (e.g.^53–55^), yet, less is known for termites. A recent study on the less socially complex termite *C. secundus* uncovered a network of co-expressed genes, the queen central module (QCM), which characterizes queens^30^. Importantly, the QCM contains genes from all major pathways known to regulate life history traits, including genes linked to JH signalling, the insulin/insulin-like growth factor1 (IIS) pathway, and fecundity. The QCM also contained the orthologues of all three Vgs that characterize *M. bellicosus* queens^43^ and which we found upregulated in builders compared to foragers. This supports our conclusion that task division reflects mechanisms underlying reproductive DOL. This provides first hints that the reproductive ground plan hypothesis seems valid for termites as well.

### Foraging aligns with deteriorating physiological conditions

As in other studies on social and solitary insects^56^, foraging in *M. bellicosus* workers seems to be associated with low Vg expression and poor physiological conditions (indicated by the presence of urocytes), though sample size is low for the latter, requiring further studies. By contrast, builders were characterized by GO terms indicating protein synthesis and high energy metabolism, including glycolytic processes.

The role of urocytes in termites seems complex and might differ between species^45^. In the termite *Reticulitermes speratus*, urocytes have been described as a reservoir of nitrogen in the form of uric acid, that are supposed to contribute to termites’ survivorship by functioning as antioxidants^46^. In *Macrotermes* species, including *M. bellicosus*, the occurrence of whitish urocytes (as urate or uric acid) has been associated with stress or old age^38,40^. As the studied workers were of similar age, age is unlikely to explain the difference in the presence of urocytes. Further support for an association with stress, at least in some species, comes from *C. secundus*. Own studies that followed termite individuals over extended periods, showed that individuals of all castes can acquire urocytes during stressful conditions, such as an infestation with mites or poor food quality. More studies are needed to understand the role of urocytes in termites.

In contrast to social Hymenoptera, however, we did not detect differences in JH titres between foragers and builders in *M. bellicosus*, yet sample size is small especially for foragers (Fig. 2). Foraging *M. bellicosus* workers are of similar age as builders. However, in studies on social Hymenoptera foraging workers are often compared with nursing workers, which naturally differ in age, if not controlled for in the experimental set up. High JH titres are known to align with ageing in solitary^57^ and social insects^23,58,59^. Hence, poor physiological conditions appear to lead to a task switch to foraging rather than JH levels. This has primarily been shown in some social Hymenoptera (e.g.^21,23^).

This may lead to a hypothesis how task division can be stably maintained through re-enforcing feedback mechanisms caused by social interactions. If low nutritional status triggers individuals to forage and if returning foragers are permanently ‘depleted’ by begging nestmates, stable task division may result. Similar ideas have been put forward^13,60,61^.

### Foragers invest less into anti-ageing mechanisms

Our DEG data support the hypothesis that an increased age-dependent extrinsic mortality aligns with fastened intrinsic ageing^41,42^. High age-dependent extrinsic mortality can select for a shortened lifespan and reduced investment into mechanisms to prevent ageing. Foragers had a lower expression of anti-oxidant genes than builders. These genes alleviate damage from oxidative stress caused by elevated ROS (reactive oxygen species) levels^62–65^.

To summarize, our results support the hypothesis that termites have co-opted similar genes as social Hymenoptera underlying task division in workers. Furthermore, these genes characterize reproductive DOL in social insects in a general manner. Additionally, foraging *M. bellicosus* workers seem to be in a worse physiological condition than similar-aged builders, as indicated, for instance, by the lower expression of the vitellogenin genes, the presence of urocytes, and the lower expression of catalases and SODs that can keep ROS levels in check. This supports the hypothesis that individual physiological state rather than intrinsic age triggers the switch to foraging. Finally, in line with predictions from life history theory that high age-dependent extrinsic mortality can select for faster intrinsic ageing (senescence), foragers seem to invest less in anti-ageing mechanisms than builders, which are less exposed to extrinsic mortality risks.

## Methods

### Transcriptome experiment

#### Sampling and generation of transcriptome data

*M. bellicosus* samples were collected in the Comoé National Park (coordinates 8°46'N 3°47'W, Côte d'Ivoire). For the transcriptome analyses, we used 14 minor workers of similar age, estimated by mandible wear, as described under ‘Generation of JH and urocyte data set’^66^. Each individual originated from a different colony to provide independent samples. Individuals were collected while performing building (n = 7) or foraging (n = 7) tasks. Transcriptomes were generated from the termites' head plus thorax. Recent studies showed that head plus thorax samples are most suitable for transcriptome studies in termites as major pathways underlying the regulation of life history traits, including the fecundity in queens, are expressed in these tissues^30,49^.

The samples were prepared and generated exactly as in our previous study from which we used the queen expression data (including sample preparation and analyses by the same person)^43^. They were then sent to BGI, Hong Kong on dry ice. Library preparation was done by BGI using the TruSeq RNA Kit following the BGI sample preparation protocol. Details for each sample can be found under Bioproject ID PRJNA727164. Amplified libraries were sequenced on an Illumina HiSeq 4000 platform with strategies of 100 bp paired-end (PE) reads, generating at least 4 Gigabases of raw data for each sample. After sequencing, index sequences from the machine reads were demultiplexed (sorted and removed) by a proprietary BGI-inhouse tool.

#### Preprocessing of RNASeq raw reads

Raw sequence reads from all samples provided by BGI were used for all further steps. After quality control with FastQC v. 0.11.9^67^, raw sequence reads were checked for a minimum length of 70 bp and adapters (including two BGI inhouse adapters) were trimmed using Trimmomatic v. 0.39^68^. Trimming and other subsequent steps were facilitated by GNU parallel v. 20141022^69^.

#### Gene expression and mapping against the genome of *Macrotermes natalensis*

We used the genome of *Macrotermes natalensis* (Haviland 1898) version 1.0 as a mapping backbone in our study^70^ which serves well for this purpose^43^. Trimmed and cleaned raw reads were mapped against the *M. natalensis* genome using the program Hisat2 v. 2.2.0 with default settings^71^. The resulting bam files were sorted by gene name using SAMtools (v. 1.5)^72^. HTSeq v. 0.9.1^73^ was then used to count the mapped raw reads per gene (stranded = no, type = gene, mode = union, order = name) for each sample. A plain text table summarizing all samples was compiled (Supplementary Table S6).

#### Gene expression analysis

DESeq2 v. 1.18.1 was used in R v. 3.4.4 to analyse differential gene expression between builders and foragers^74,75^. To test for pairwise expression differences, we used the generalized linear model with negative binomial distribution as implemented in DESeq2. We compared differential gene expression between two tasks, builders and foragers (Supplementary Tables S1–S2, S4). FDR-adjusted p-values (padj) were used to correct for multiple testing^76^. To analyse the similarities between builder/forager and queen gene expression, we compared the overlap of the DEG lists: We compared genes that were upregulated in queens compared to all other castes from a previous study^43^ to the genes upregulated in builders compared to foragers from the current study (Fig. 1). Similarly, we compared the overlap between queen genes and those upregulated in foragers compared to builders (Fig. 1).

#### Functional annotation and GO analysis

Functional annotation of the amino acid (AA) sequences of protein coding *M. natalensis* genes was done in Interproscan v. 5.41-78.0^77^. The databases searched were pfam, panther, cdd, coils, gene3d, hamap, pirsf, prints, sfld and superfamily. We extracted the GO annotation for each AA sequence and combined entries from all databases for each. We used NCBI BLASTX v. 2.2.31+ to compare the coding sequences of the *M. natalensis* genome with the NCBI nr protein database (accessed Nov. 2015)^78^. We applied TopGO v. 2.30.1^79^ to compare builder- and forager-genes; we examined the GO hierarchy and overrepresented terms and built graphical representations. We analysed Biological Process (BP), Cellular Component (CC) and Molecular Function (MF) (Supplementary Table S3).

### Generation of JH and urocyte data set

*M. bellicosus* individuals were collected in the field in the Comoé National Park (see above). The quantity of urocytes was estimated in the field as high or low according to the obvious presence or absence of large urocytes. They are visible as white ‘crystals’/dots in the fat body through the termites’ transparent cuticle. From these same individuals haemolymph samples were taken for JH measurements. To do this, their abdomens were punctured with a pin, and protruding droplets of haemolymph were absorbed into calibrated glass capillaries (5 µL single-use-capillary pipettes, Neolab, Hirschmann, Eberstadt, Germany) (for more details see^35^). For JH measurements, haemolymph aliquots of single individuals of 1 µL were immediately dissolved in 0.5 mL acetonitrile. These samples were stored at -20 °C (except for transport from the field to the laboratory) in Teflon-stoppered screw-cap glass vials. The remaining dead individuals were stored in ethanol for age determination in the lab by studying mandible wear under a dissecting microscope. Hence, we only knew in hindsight the age of the individuals and we had to reject samples, which were too old (very worn mandibles; mainly foragers) or too young (largely unused mandibles; a few builders). This reduced and biased our sample size so that we had in the end haemolymph samples from 14 old building minor workers and 3 old foraging minor workers. Each individual originated from a different colony to provide an independent sample.

JH was extracted following a liquid-phase separation protocol^80^, as described in^35^. The RIA protocol, including dilutions of the JH-specific antiserum, radioactivity concentration of [10 ^−^^3^H(n)]-juvenile hormone III (specific activity 19.4 Ci/mmol Perkin Elmer Life Sciences, Waltham, MA), and the standard curve range (50–10 ng) established with unlabelled juvenile hormone III (Fluka, Buchs, Switzerland), in the same way as in our prior study^35^. JH titres of the samples were calculated based on a non-linear four-parameter regression on standard curve values (ImmunoAssay Calculations spreadsheet, Bachem, Bubendorf, Switzerland) and are expressed as JH-III equivalents (pg/μL hemolymph). JH-III is the only JH moiety produced by termite corpora allata and detected in the haemolymph^34,81^.

The urocytes and JH data sets were analysed using non-parametric statistics. We compared the frequency of the occurrence of urocytes between foragers and builders using contingency analysis, while the quantity of JH titres between foragers and builders was analysed with Mann Whitney U-test. All tests were two-tailed and statistical analyses were done with IBM SPSS 23.0^82^. We tested the power of these using G*Power 3.1.9.7^83,84^.

## Supplementary Information

Supplementary Information 1.

Supplementary Information 2.

## Data Availability

The raw sequence data used in this publication has been deposited in the NCBI SRA archive under the accession PRJNA727164. Data used to generate figures are provided with this paper.
