# Supplementary material for: Molecular underpinnings of division of labour among workers in a socially complex termite
Source: Sci Rep. 2021 Sep 14;11:18269. doi: 10.1038/s41598-021-97515-w (PMC8440649; doi:10.1038/s41598-021-97515-w)
Supplement: Supplementary file 2 — Supplementary Information 2. [file 41598_2021_97515_MOESM2_ESM.docx]

Supplementary Material for: Molecular underpinnings of division of labour among workers in a socially complex termite

Daniel Elsner^1^, Klaus Hartfelder^2^, Judith Korb^1.*^

^1^Evolutionary Biology & Ecology, University of Freiburg, Freiburg, Germany

^2^Faculdade de Medicina de Ribeirão Preto, Universidade de São Paulo, Ribeirão Preto, Brazil

*Corresponding Author: Judith Korb: judith.korb@biologie.uni-freiburg.de

Supplementary Results

Supplementary Tables are provided as separate Excel-Files:

Supplementary Table S1 Genes upregulated in builders compared to foragers

Supplementary Table S2 Genes upregulated in builders compared to foragers, filtered by log2FC > 2

Supplementary Table S3 All GO terms that were significantly upregulated

Supplementary Table S4 Genes upregulated in foragers compared to builders

Supplementary Table S5 Juvenile hormone titres and presence of urocytes

Supplementary Table S6 Gene expression counts table

# Supplementary Figures: Gene Ontology (GO) analysis

 Supplementary Figure S1: Biological Process terms overrepresented in builders. TopGO Network Graph generated with the standard TopGO algorithm.

Supplementary Figure S2: Cellular Component terms overrepresented in builders. TopGO Network Graph generated with the standard TopGO algorithm.

Supplementary Figure S3: Molecular Function terms overrepresented in builders. TopGO Network Graph generated with the standard TopGO algorithm.

Supplementary Figure S4: Biological Process terms overrepresented in foragers. TopGO Network Graph generated with the standard TopGO algorithm.

Supplementary Figure S5: Cellular Component terms overrepresented in foragers. TopGO Network Graph generated with the standard TopGO algorithm.

Supplementary Figure S6: Molecular Function terms overrepresented in foragers. TopGO Network Graph generated with the standard TopGO algorithm.
